# Quantifying and filtering knowledge generated by literature based discovery

**DOI:** 10.1186/s12859-017-1641-9

**Published:** 2017-05-31

**Authors:** Judita Preiss, Mark Stevenson

**Affiliations:** Department of Computer Science, University of Sheffield, Regent Court, 211 Portobello, Sheffield, UK

**Keywords:** Data mining, Literature based discovery in the biomedical domain, Biomedical text

## Abstract

**Background:**

Literature based discovery (LBD) automatically infers missed connections between concepts in literature. It is often assumed that LBD generates more information than can be reasonably examined.

**Methods:**

We present a detailed analysis of the quantity of hidden knowledge produced by an LBD system and the effect of various filtering approaches upon this. The investigation of filtering combined with single or multi-step linking term chains is carried out on all articles in PubMed.

**Results:**

The evaluation is carried out using both replication of existing discoveries, which provides justification for multi-step linking chain knowledge in specific cases, and using timeslicing, which gives a large scale measure of performance.

**Conclusions:**

While the quantity of hidden knowledge generated by LBD can be vast, we demonstrate that (a) intelligent filtering can greatly reduce the number of hidden knowledge pairs generated, (b) for a specific term, the number of single step connections can be manageable, and (c) in the absence of single step hidden links, considering multiple steps can provide valid links.

## Background

Between 2,000 and 4,000 articles are added to PubMed, the National Library of Medicine’s (NLM) database of publications in biomedicine, every day [1]. This forces researchers to specialize in narrower aspects of their field and they may miss inferable connections, for example ones that reveal new treatments for diseases (e.g. Swanson [2] automatically discovered a previously unnoticed connection between *fish oil* and *Raynaud disease*, via a number of terms such as *blood viscosity*, *platelet aggregation*, *vascular reactivity*, a connection which was later verified [3]). Literature based discovery (LBD) automates the process of finding new connections (hidden connections) between existing knowledge, and thus can be used for disease candidate gene discovery, to find other uses of existing drugs, or for drug side effect prediction [4].

**Fig. 1:**
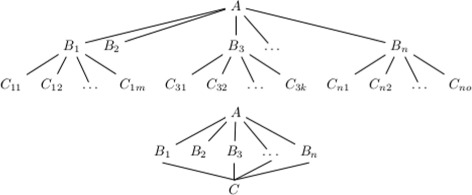
*Top*: open discovery (only *A* specified), *bottom*: closed discovery (both *A* and *C* specified)

In the frequently used *A-B-C* model [2], LBD proposes a hidden connection between two previously unconnected terms, *A* and *C*, if there is a document linking *A* to some term *B* and the same *B* is linked to *C* elsewhere. Clearly, in open discovery where only *A* is specified (shown at the top of Fig. 1), the quantity of hidden connections suggested rises with input and so LBD systems frequently grossly restrict scale. When system execution and evaluation is not restricted to a toy example, numerous output reductions are put in place, including filtering of terms (whether by discarding uninformative terms or restricting terms to, say, diseases and treatments only), restricting either the time period from which hidden knowledge is generated or the segment of the abstract that knowledge is drawn from (e.g. titles only) and re-ranking of the subsequently produced hidden knowledge, often targeted to the search for a specific discovery or type of discoveries.

Without filtering, large scale LBD becomes computationally difficult and the resulting hidden knowledge can be practically unusable. To give an idea of the scale, consider the frequently used approach using title word co-occurrence as an indication of relatedness (i.e. requiring one title to contain *Raynaud disease* and *blood viscosity* and another to contain *blood viscosity* and *fish oil* to propose a connection between *Raynaud disease* and *fish oil*): there are over 92,000 distinct words in titles of PubMed articles between 1700 and 2005, giving rise to over 561,000 co-occurring pairs. Clearly, this will give rise to a large amount of hidden knowledge if these co-occurrences are all followed, making it impossible for all hidden knowledge to be explored. Therefore some filtering is required, however, it is crucial that important links or terms are not removed. Previously explored filtering options include:

1. **Time period**. Much of earlier LBD work restricted the knowledge base to a reduced time segment – for example, Gordon and Lindsay [5] restricted publications to the years 1983–1985 when they sought to replicate Swanson’s [2] *fish oil* – *Raynaud disease* connection.
2. **Relation**. Preiss et al. [6] show that employing more sophisticated definitions of links between terms (relations) greatly reduces the number of hidden knowledge pairs generated without detrimental effect on performance.
3. **Stoplist**. For example, Swanson et al. [7] start by removing non-content words and add, semi-automatically, other terms to a growing stoplist (in 2006, this contained 9,500 terms).
4. **Literature reduction**. Swanson et al. also carry out term reduction at an earlier stage: they pre-filter the literature on a per sought term basis by subject heading. If the user is seeking term *X*, hidden knowledge is only generated from abstracts which contain *X* in their MeSH subject heading and where *X* is present in the title. (Note that this clearly requires prior knowledge of search terms.)
5. **Term type**. Yetisgen-Yildiz and Pratt [8] limit the types of linking and target terms permitted (to categories such as *chemicals & drugs* or *genes & molecular sequence*) on the basis that this is the type of link they wish to find.
6. **CUIs vs terms**. The Unified Medical Language System Metathesaurus (UMLS) [9] is a large thesaurus which lists millions of biomedical and health related concepts using Concept Unique Identifiers (CUIs). Weeber et al. [10] filter out non-content words by switching from terms to UMLS CUIs. Aside from removing non-content words, switching to CUIs also avoids spurious connections due to term ambiguity. To identify the correct CUIs, they use MetaMap [11], a publicly available tool which assigns UMLS CUIs to terms, as well as mapping words to multi-word units where appropriate.
7. **Synonym merging**. While not carrying out explicit synonym merging, Cameron et al. [12] manually add close terms to the source (*A*) and target (*C*) term in closed search (see bottom of Fig. 1) LBD. As both *Raynaud disease* and *Raynaud phenomenon* appear separately in UMLS, the hidden knowledge generated will vary if these are treated as one unit.
8. **Relation type**. Focusing on one type of discovery, adverse drug reactions, Shang et al. [13] employ only the INTERACTS_WITH and COMPARED_WITH relations within one step of their inference process.

The *A*−*B*−*C* model generates justification(s) for each hidden connection, the linking (*B*) terms – *raynaud disease* and *fish oil* were found to be connected via the linking term *blood viscosity*, with one decreasing and the other increasing the same. However, hidden knowledge can be identified by following longer paths of linking terms, i.e. *A*→*b*_1_→*b*_2_→⋯→*b*_*n*_→*C*. This approach shows promise and has already been explored. For example, Kontostathi and Pottenger [14] investigate paths of linking terms generated by co-occurrence and Wilkowski et al. [15] show the feasibility of the approach on a single *A* and *C* pair. However, previous evaluation of this approach has been restricted to small numbers of examples and no large scale evaluation has yet been carried out.

The novelty of our work lies in a detailed analysis of the quantity of hidden knowledge produced and the effect of various filtering approaches upon this. This thorough investigation of filtering combined with single or multi-step linking term chains is, to our knowledge, the first comprehensive investigation of this type.

## Methods

### Literature based discovery system

We use an LBD system which accepts an adjacency matrix *M* describing relations between pairs of terms in a term collection: the entry *m*_*ij*_ is a positive integer if a relation *R* is detected between terms *t*_*i*_ and *t*_*j*_. If *t*_*i*_ and *t*_*j*_ are not directly related anywhere in the document collection, *m*_*ij*_ will be zero. Using graph theory [16], any non zero terms in

$$\text{norm}(M^{2}) - \text{norm}(M) $$

where norm converts *m*_*ij*_ to 1 if *m*_*ij*_>0 and leaves 0 otherwise, represent connections via one linking step. The system can be extended to find connections via any number of linking steps, for example any positive (non zero and non negative) terms in

$$\text{norm}(M^{3}) - \text{norm}(M^{2}) - \text{norm}(M) $$

represent connections via two linking steps. Similarly, connections via three steps can be obtained and so on.

### Relations

The LBD system described above relies on the existence of a relation between a pair of terms. We base our relations on the output of the SemRep system [17] which uses underspecified syntactic processing and UMLS [9] domain knowledge to extract subject-relation-object triples (such as *X-treats-Y* or *X-affects-Y*) from biomedical texts. Building on the output of MetaMap [11], SemRep extracts a number of positive and negative relations as well as a positive and negative comparative relations. For example, from the sentence in 1 SemRep extracts the relations in 2 (terms are presented here for ease of understanding; SemRep extracts CUIs rather than terms):

1. We used hemofiltration to treat a patient with digoxin overdose that was complicated by refractory hyperkalemia.
2. Hemofiltration-TREATS-Patients
  Digoxin overdose-PROCESS_OF-Patients
  hyperkalemia-COMPLICATES-Digoxin overdose
  Hemofiltration-TREATS(INFER)-Digoxin overdose

A SemRep processed version of Medline is available from NLM [18], and we use all positive relations from semmedVER24_2 processed up to 30 June 2014 (which contains 70,364,020 relations) to populate the adjacency matrix *M*.

A clear advantage of SemRep is its output in ‘UMLS CUI – relation - UMLS CUI’ format: this excludes non-content words, as CUIs do not exist for these, and ensures that a hidden connection is found via a compatible sense of a term. For example, the top five UMLS senses of *cold* are: cold temperature, common cold, cold therapy, chronic obstructive lung disease and cold sensation. If SemRep did not yield CUIs, its output would be:

- Mechanical ventilators TREATS Chronic obstructive lung disease
- Common cold PROCESS_OF Rhinovirus

If the two senses of *cold* were not differentiated, a hidden connection could be found between *mechanical ventilators* and *rhinovirus*.

While mapping to CUIs clearly reduces the number of incorrect hidden knowledge pairs, it will not eliminate connections via general terms – words such as *patient*, *clinical study* or *week*. The following section discusses a number of filtering techniques.

### Filtering

#### sy - synonym merging

As with any type of dictionary, a decision is made by the creators as to dividing up senses, termed as lumping or splitting in lexicography [19]. As UMLS is composed of terms from multiple source vocabularies, the splitting / lumping decision is not consistent throughout. In general, there is a tendency to split senses and later merge based on application, and we therefore, based on finding separate CUIs for *Raynaud disease* and *Raynaud phenomenon* and finding that Cameron et al. [12] manually augment their selected *A* and *C* terms “with related concepts”, investigate an automatic approach to synonym merging within UMLS.

Note that merging synonyms will affect the quantity of *A* and *C* terms as well as possible linking terms, *B*:

- If the start point, *A* (e.g. *Raynaud Disease*), has multiple synonymous CUIs, merging these will result in the generation of more hidden knowledge from *A*.
- If a linking term CUI is equivalent to the CUI of another term, there could be more hidden knowledge generated. Examination of documents supporting a *Raynaud disease – fish oil* link reveals that some of the expected connections from CUI C0034734 (*Raynaud disease*) are linked to CUI C0034735 (*Raynaud phenomenon*) instead. Although the two terms are synonymous for the purposes of evaluating the *Raynaud disease – fish oil* link, due to their different CUIs, the connection will not be found.
- Since synonymous pairs of hidden knowledge (and linking terms) will merge, this will reduce the burden on a user.

The synonymous, SY, relation within UMLS is source asserted synonymy, and thus is listed alongside a source. The quality of synonyms in UMLS has been questioned [20], and we evaluate the synonym classes created (these are formed by gathering all synonyms, and their synonyms etc., into disjoint classes) for synonyms asserted by one or more sources. Table 1 displays details of the synonym information broken down by the minimum number of sources supporting each extracted SY relationship (the first column), with the second column representing the number of synonym classes with at least 2 distinct CUIs. The remaining columns describe the synonym classes: the largest synonym class (max), the number of synonym classes containing at least *X* CUIs (>*X*) and the mean synonym class size.

**Table 1:** Information about synonym classes related to the number of sources supporting the synonymy

Min sources	Num classes	Max	>20	>10	>7	>5	>3	Mean
1	11,030	74	21	174	401	850	1,938	2.91
2	1,542	73	1	1	3	5	26	2.14
3	6	3	0	0	0	0	0	3

Basing classes on 1 source produces a class of 74 elements – this is unlikely to contain synonyms useful for knowledge discovery. Synonym classes with more than 20, or even 10, elements are similarly unlikely to be helpful: we employ synonym classes supported by at least two sources with class size ≤5 which leaves 614 synonym classes and reduces the original 2,868,943 UMLS CUIs to 2,867,188 distinct CUIs (restricting to CUIs that appear in SemRep relations in Medline, this reduces the original 485,538 CUIs to 484,924 distinct CUIs).

#### st - semantic type

The UMLS Semantic Network contains a hierarchy of subject categories, semantic types (STs), with at least one assigned to each CUI. Previous work selects a number of STs allowed to act as linking or target terms as these are thought to describe the type of desired hidden knowledge; for example Yetisgen-Yildiz and Pratt [8] allow both linking and target terms to be *chemicals & drugs* and *genes & molecular sequences*, but linking terms can also be *disorders*, *physiology* and *anatomy members*.

Rather than restricting CUIs to a small number of STs according to the type of discovery expected (which requires prior knowledge), we explore a number of general ST exclusions:

- **Obvious** removes sts which rarely appeared in useful relations: activities & behaviours, geographic areas, occupations, organizations and procedures.
- **Manual** based on the expert opinion of the likelihood of being in relations, 70 sts were manually selected for exclusion [21].
- **Half** contains the st supertypes for which at least half of the subtypes were removed in *manual*: activities & behaviours, geographic areas, occupations, organizations, procedures, anatomy, concepts & ideas, devices, living beings and objects.

And one inclusion approach:

- **Y-Y&P** containing the sts chemicals & drugs, genes & molecular sequences, disorders, physiology and anatomy members.

**Fig. 2:**
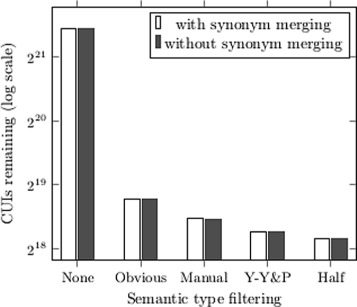
CUIs remaining after removing semantic types

Figure 2 shows the decrease in number of CUIs when various semantic types are removed.

#### clt - common linking terms stoplist

**Fig. 3:**
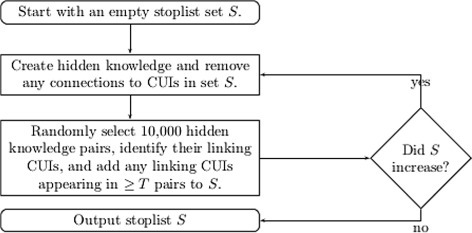
Pseudocode for creating a stoplist by identifying common linking terms

Some CUIs correspond to terms which are clearly too general but their ST also contains useful CUIs and therefore should not be removed. Although UMLS is hierarchically structured, and thus general terms could be expected closer to root nodes, it is composed of multiple hierarchies with different levels of granularity and so an overall threshold is unlikely to be found. The hypothesis that a CUIs which frequently acts as a linking term is unlikely to be informative gives rise to an automatic technique for building a stoplist shown in Fig. 3 [22]. We create our stoplist from the 1865–2000 segment of Medline.

#### break – breaking common linking term connections

A filtering technique based on a stoplist needs to be quite conservative so it does not remove useful terms and therefore it is likely to leave some unhelpful terms. Breaking common linking term connection is a fundamentally different idea to creating a stoplist: instead of finding frequently appearing terms, this approach bases its decisions on the number of terms a given term is connected to.

Terms *A* and *B* are related if a (non negative) SemRep relation exists between them. An uninformative word, such as *study* or *patient*, can be expected to be connected to a large number of CUIs. The hypothesis that highly connected terms are likely to be fairly general (and therefore not useful linking terms), gives rise to the following filtering options:

1. When creating the matrix A, break (discard) all connections to CUI *A* when the *C*(*A*)>threshold.
2. Discard the connection between CUIs *A* and *B* when min(*C*(*A*),*C*(*B*))>threshold.

(Where *C*(*A*) represents the number of CUIs linked to *A*, and the threshold needs to be empirically determined.)

## Results and discussion

LBD is clearly difficult to evaluate: by virtue of the generated knowledge being new, there is no gold standard for comparison. Two standard techniques for evaluation exist: 1) replication of existing discoveries (e.g. [5, 10, 23]), where discoveries made using previous LBD systems are collected from literature and a new LBD system is employed over the same time segment in an attempt to produce the same discovery, and 2) timeslicing [24], which allows the generation of precision and recall figures by allowing a gold standard to be automatically created from publications after a cut off date with hidden knowledge generated from publications prior. We present both types of evaluation below.

### Replication of existing discoveries

Seven separate discoveries were identified from LBD literature which have previously been used for replication experiments. We include the time segment used in the original discovery and remove any documents containing a direct link between the *A* and *B* terms – this can be present for a number of reasons a) LBD is being employed to suggest alternatives, e.g. alternative treatments, or b) the connection was removed in previous work, for example due to a manual inspection showing that *A* and *B* are not related despite co-occurring in the same title – the number of documents removed is described as the number of direct connections and the abbreviation used in Table 2 is also included:

1. [**RD-fsh**] *Raynaud disease* – *fish oil* [5, 10, 25]; 1960–1985, no direct connections.

**Table 2:** Number of linking terms yielded in replication of existing discoveries

	Mig–Mg	RD–fsh	Som–Arg	Mg–ND	AD–est	Sc–iPL	AD–INN
Number of linking terms found after a single step							
unfiltered	81	2	232	101	490	0	365
sy	76	2	221	97	485	0	356
manual	58	0	157	57	362	0	258
Y-Y&P	0	2	0	1	1	0	0
clt	54	0	147	55	350	0	248
break	47	0	114	38	0	0	0
Number of linking terms found after two steps							
unfiltered	49,877	1,386	132,514	69,669	424,712	9	386,098
sy	46,551	1,333	126,178	65,845	408,875	9	371,354
manual	25,921	510	67,206	27,046	217,834	9	203,616
Y-Y&P	0	602	44	121	396	0	0
clt	23,258	453	59,317	25,227	200,720	8	187,936
break	17,659	361	35,323	11,173	0	8	0
2. [**Som-Arg**] *Somatomedin C* – *arginine* [26]; 1960–1989, 27 direct connections.
3. [**Mig-Mg**] *Migraine disorders* – *magnesium* [25]; 1980–1984, no direct connections.
4. [**Mg-ND**] *Magnesium deficiency* – *neurologic disease* [27]; 1960–1994, no direct connections.
5. [**AD-INN**] *Alzheimer’s disease* – *indomethacin* [28]; 1966–1996, 6 direct connections.
6. [**AD-est**] *Alzheimer’s disease* – *estrogen* [29]; 1960–1995, 25 direct connections.
7. [**Sc-iPL**] *Schizophrenia* – *Calcium-Independent Phospholipase A2* [30]; 1960–1997, 1 direct connection.

**Fig. 4:**
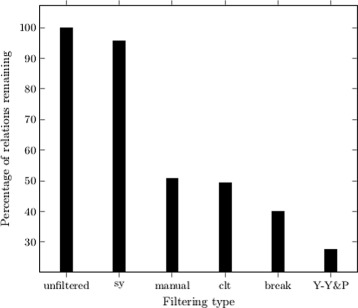
Percentage of original relations remaining after filtering

Figure 4 shows the effect that the various filtering algorithms have on the number of SemRep relations remaining within each discovery’s segment (the filtered number of direct connections remaining is averaged over the 7 discoveries and the percentage remaining, in comparison to the original, unfiltered set, is presented). Except for the unfiltered, original, results, all other filtering results carry out synonym merging, *clt* and *break* are added on top of manual semantic type filtering. The graph shows that filtering is an effective way of reducing the number of relations: with the exception of synonym merging alone, all filtering approaches reduce the number of direct relation pairs by at least 50%.

Table 2 presents the number of linking terms found when each discovery is replicated: the upper part of the table describes the number of linking terms corresponding to single step connections. Since the single step approach sometimes fails to find a connection, two step connections are sought and are presented in the lower part of the table. The number of linking terms generated for each discovery is clearly linked to the number of connections input – however, despite the connection being inferrable using co-occurence, the reduction to zero hidden links using SemRep combined with filtering is valid. For example, the two linking terms connecting *Raynaud disease* to *fish oil* with synonym merging are C0029064 (operating theatre) and C0040426 (set of teeth). Neither clearly supporting the connection and therefore justifiably removed with semantic types.

It is interesting that in most filtering cases, the frequently cited *Raynaud disease* – *fish oil* connection is not replicated: this was revealed to be due to a combination of synonym failure and the relation employed not extracting the necessary connections. The *schizophrenia* – Ca^2+^iPLA2 link is not replicated via a single step as Ca^2+^iPLA2 only appears very few times in Medline and is only seen in one SemRep relation. In these cases, it is worth examining two step connections:

#### schizophrenia – Ca^2+^iPLA2

The 9 two step connections for schizophrenia (C0036341)– Ca^2+^iPLA2 (C0538273) generated with *manual* filtering can be seen below:

1. C0001473 (atpase) – C0020063 (Parathyroid Hormone)
2. C0001655 (adrenocorticotropic hormone) – C0020063 (Parathyroid Hormone)
3. C0003779 (Arginine vasopressor) – C0020063 (Parathyroid Hormone)
4. C0021641 (Regular insulin) – C0020063 (Parathyroid Hormone)
5. C0021740 (Recombinant Interferon Gamma) – C0020063 (Parathyroid Hormone)
6. C0033371 (Prolactin preparation) – C0020063 (Parathyroid Hormone)
7. C0037659 (Somatostatin preparation) – C0020063 (Parathyroid Hormone)
8. C0040160 (Thyrotrophin product) – C0020063 (Parathyroid Hormone)
9. C0041249 (tryptophan (Trp)) – C0020063 (Parathyroid Hormone)

As the UMLS definition states, the parathyroid hormone elevates blood Ca2+ levels and thus is related to the *PLA2G6* protein, showing all nine connections to be worthy of further consideration.

#### fish oil – Raynaud disease

For *fish oil* and *Raynaud disease*, the two step connections include:

1. C0005823 (blood pressure) - C0006938 (captopril)
2. C0005848 (blood viscosity) - C0030899 (pentoxyphylline)
3. C0005848 (blood viscosity) - C0232338 (blood flow function)
4. C0005848 (blood viscosity) - C0206502 (hemorheology)

Captopril and pentoxyphylline are used in the treatment of Raynaud’s phenomenon, and both Raynaud’s and fish oil are known to affect blood viscosity. The blood viscosity links are also supported by the linking term analysis in [10].

These findings suggest that multi linking term exploration is worth pursuing when a connection is suspected but is not found via a single step connection.

### Timeslicing

One of the drawbacks of evaluating LBD by replicating existing discoveries is that it relies on the use of small test sets. Timeslicing is an alternative approach that allows larger test collections to be created automatically. A cutoff date is chosen, hidden knowledge is generated from publications published prior to this date and the resulting pairs are compared to the new knowledge published after the cutoff (as identified by the used relation) [24]. This section reports results using the timeslicing approach.

Hidden knowledge is generated from all publications listed in Medline up to the end of 2005 and it is evaluated against a gold standard generated from 2006–2015. The gold standard is created by extracting all SemRep relations from abstracts published after the cutoff and removing any SemRep pairs present in Medline before the cutoff; this leaves 1,193,495 pairs.

The results of a timeslicing evaluation are presented in Table 3 – these include the total number of pairs of hidden knowledge generated, the number of generated pairs which appear in the gold standard (“correct”), the precision (the percentage of pairs generated that are in the gold standard), recall (the percentage of pairs present in the gold standard that were generated) and the F-measure, a combination of precision and recall. Again, the upper part of the table represents results for single step connections, and the lower part represents both one and two step connections. There is an obvious trade off between precision and recall – the higher the number of pairs returned, the greater the recall, but obviously the less likely it is for a person to be able to go through the resulting knowledge: the last column lists the average number of pairs of hidden knowledge generated per term seen in the segment.

**Table 3:** Timeslice evaluation

Filtering	Total	Correct	Precision	Recall	F-measure	Average
Performance after a single step						
sy	1,049,250,170	526,363	0.05	44.10	1.00e-03	11,131
manual	386,952,997	268,327	0.07	22.48	1.38e-03	6,099
Y-Y&P	243,218,893	190,072	0.08	15.93	1.56e-03	4,952
clt	387,603,836	269,003	0.07	22.54	1.38e-03	6,103
break	131,199,050	213,193	0.16	17.86	3.22e-03	2,232
Performance after two steps						
sy	3,733,002,802	534,301	0.01	44.77	2.86e-04	39,602
manual	1,638,685,466	274,544	0.02	23.00	3.35e-04	25,828
Y-Y&P	994,744,004	194,749	0.02	16.32	3.91e-04	20,257
clt	1,641,987,567	275,230	0.02	23.06	3.35e-04	25,857
break	1,085,230,979	227,998	0.02	19.10	4.20e-04	18,467

The overall precision can be expected to be low for a number of reasons, including:

1. Even if the hidden knowledge generated is genuine, it may not have been discovered yet within the segment on which the gold standard is based.
2. Some knowledge will be known but never published as it is considered ‘obvious’. An LBD system will generate such knowledge nonetheless.

While the total amount of hidden knowledge generated may seem unmanageable and not useful, it is supported by manual findings: UMLS includes manually identified relations, and on average goes through 2 releases per year. There were 29,936,977 instances of relations added between 2015AA and 2005AA version of UMLS. Since 29,936,977×4=119,747,908 (with the number of hidden knowledge pairs generated using *break* =131,199,050), the quantity of hidden knowledge produced no longer seems unreasonable.

In turn, low precision accounts for the low F-measure; again, this is not unusual for large scale timeslicing results in LBD [6]. The highest F-measure for single step connections (3.22e-03) is achieved by breaking common linking term connections. In this setting the amount of hidden knowledge generated (an average of 2,232 pieces per term) is much more manageable than the amount generated without filtering (34,986 pieces per term).

The very low recall (and precision) of the two step connections is caused by the high recall from the one step connections which results in a large number of two step connections to be generated (see Literature based discovery system).

The large number of two step connections will, in general, make the results unusable for open discovery. However, two step connections provide a good backoff for closed discovery when a link is suspected by cannot be found using a single step. This appears to be more common with rarer, thus more likely to be specific, concepts (such as Ca^2+^iPLA2).

The results do raise the question of whether precision and recall are good measures for evaluating large scale LBD systems.

## Conclusions

We present an extensive discussion of filtering within literature based discovery, and show that using a more sophisticated definition of relation as well as UMLS CUIs (rather than terms directly) is insufficient in itself to yielding usable quantities of hidden knowledge. We explore a number of different approaches and show their effect on both replication and timeslicing evaluations. We find the best performance from the rarely used approach which breaks connections on a term pair basis, rather than removing entire terms.

Based on the results of replication of existing discoveries, we propose that the quantity of hidden knowledge generated for a term *A* will be proportional to its overall frequency within the corpus, and argue that the high proportion of frequent terms is the cause of the low F-measure found using timeslicing evaluation. A comparison with the number of relations (manually) added to UMLS on each release also suggests a high expected number of hidden knowledge pairs.

We also examine the possibility of generating hidden knowledge between terms *A* and *C* using a chain of multi-step linking terms *b*_*i*_, i.e. *A*→*b*_1_→*b*_*n*_→*C* with no direct connection between *A* and *C*. While such an approach clearly generates an unmanageable quantity of data in open mode, its value can be seen when a single step connection fails to be found in closed mode: in this case, some multi step connections may be suggested and we propose using a multi step system as a backoff for failed single connection LBD.

## References

[1] NLM: MEDLINE fact sheet. https://www.nlm.nih.gov/pubs/factsheets/medline.html. Accessed: 2017-03-17.

[2] Swanson DR (1986). Fish oil, Raynaud’s syndrome, and undiscovered public knowledge. Perspect Biol Med.

[3] DiGiacomo RA, Kremer JM, Shah DM (1989). Fish-oil dietary supplementation in patients with Raynaud’s phenomenon: a double-blind, controlled, prospective study. Am J Med.

[4] Hristovski D, Rindflesch T, Peterlin B (2013). Using literature-based discovery to identify novel therapeutic approaches. Cardiovasc Hematol Agents Med Chem.

[5] Gordon MD, Lindsay RK (1996). Toward discovery support systems: a replication, re-examination, and extension of swanson’s work on literature-based discovery of a connection between Raynaud’s and fish oil. J Am Soc Inform Sci.

[6] Preiss J, Stevenson M, Gaizauskas R (2015). Exploring relation types for literature-based discovery. J Am Med Inform Assoc.

[7] Swanson DR, Smalheiser NR, Torvik VI (2006). Ranking indirect connnections in literature-based discovery: The role of medical subject headings. J Am Soc Inform Sci Technol.

[8] Yetisgen-Yildiz M, Pratt W, Bruza P, Weeber M (2009). Evaluation of literature-based discovery systems. Literature-Based Discovery.

[9] Bodenreider O (2004). The unified medical language system (UMLS): integrating biomedical terminology. Nucleic Acids Res.

[10] Weeber M, Vos R, Klein H, de Jong-van den Berg LTW (2001). Using concepts in literature-based discovery: Simulating Swanson’s Reynaud – fish oil and migraine – magnesium discoveries. J Am Soc Inform Sci Technol.

[11] Aronson AR, Lang FM (2010). An overview of MetaMap: historical perspective and recent advances. J Am Med Inform Assoc.

[12] Cameron D, Kavuluru R, Rindflesch TC, Sheth AP, Thirunarayan K, Bodenreider O (2015). Context-driven automatic subgraph creation for literature-based discovery. J Biomed Inform.

[13] Shang N, Xua H, Rindflesch TC, Cohen T (2014). Identifying plausible adverse drug reactions using knowledge extracted from the literature. J Biomed Inform.

[14] Kontostathis A, Pottenger WM (2006). A framework for understanding latent semantic indexing (LSI) performance. Inform Process Manage.

[15] Wilkowski B, Fiszman M, Miller CM, Hristovski D, Arabandi S, Rosemblat G, Rindflesch TC. Graph-based methods for discovery browsing with semantic predications. In: AMIA Annual Symposium Proceedings. Washington: 2011. p. 1514–1523. PMC3243228 22195216

[16] Godsil C, Royle G (2001). Algebraic Graph Theory.

[17] Rindflesch TC, Fiszman M (2003). The interaction of domain knowledge and linguistic structure in natural language processing: interpreting hypernymic propositions in biomedical text. J Biomed Inform.

[18] NLM: Semrep. http://semrep.nlm.nih.gov/. Accessed: 2017-03-17.

[19] Atkins BTS, Rundell M (2008). The Oxford Guide to Practical Lexicography.

[20] Fung KW, Hole WT, Nelson SJ, Srinivasan S, Powell T, Roth L (2005). Integrating SNOMED CT into the UMLS: An exploration of different views of synonymy and quality of editing. J Am Med Inform Assoc.

[21] Preiss J. Excluded semantic types. http://kdisc.rcweb.dcs.shef.ac.uk/data/filtered_semtypes.txt. Accessed: 2017-03-17.

[22] Preiss J. Seeking informativeness in literature based discovery. In: Proceedings of BioNLP 2014. Baltimore: 2014. p. 112–7.

[23] Srinivasan P (2004). Generating hypotheses from MEDLINE. J Am Soc Inform Sci Technol.

[24] Yetisgen-Yildiz M, Pratt W (2009). A new evaluation methodology for literature-based discovery. J Biomed Inform.

[25] Hu X, Zhang X, Yoo I, Zang Y. A semantic approach for mining hidden links from complementary and non-interactive biomedical literature. In: SDM. Bethesda: 2006. p. 200–9.

[26] Swanson DR (1990). Somatomedin c and arginine: Implicit connections between mutually isolated literatures. Perspect Biol Med.

[27] Smalheiser NR, Swanson DR (1994). Assessing a gap in the biomedical literature: Magnesium deficiency and neurologic disease. Neurosci Res Commun.

[28] Smalheiser NR, Swanson DR (1996). Indomethacin and Alzheimer’s disease. Neurology.

[29] Smalheiser NR, Swanson DR (1996). Linking estrogen to Alzheimer’s disease. Neurology.

[30] Smalheiser NR, Swanson DR (1997). Calcium-independent phospholipase A2 and schizophrenia. Arch Gen Psychiatr.

